# Malnutrition in sickle cell anemia: Prevalence, impact, and interventions: A Review

**DOI:** 10.1097/MD.0000000000038164

**Published:** 2024-05-17

**Authors:** Emmanuel Ifeanyi Obeagu, Getrude Uzoma Obeagu

**Affiliations:** a Department of Medical Laboratory Science, Kampala International University, Uganda; b School of Nursing Science, Kampala International University, Uganda.

**Keywords:** dietary interventions, food insecurity, health outcomes, impact on health, malnutrition, prevalence, sickle cell anemia

## Abstract

Sickle Cell Anemia (SCA) is a hereditary hemoglobinopathy characterized by chronic hemolytic anemia, vaso-occlusive events, and a wide range of clinical complications. Malnutrition, often an underexplored aspect of this complex condition, plays a critical role in disease management and overall patient well-being. This publication provides a comprehensive review of the prevalence, impact, and interventions related to malnutrition in individuals with SCA. A thorough literature review reveals the multifaceted challenges faced by SCA patients in maintaining adequate nutrition. The pathophysiology of SCA, involving chronic inflammation, oxidative stress, and hypermetabolism, contributes to increased nutritional requirements and altered dietary patterns. Factors such as reduced appetite, nutrient malabsorption, dietary restrictions, and socioeconomic disparities further exacerbate the risk of malnutrition. Malnutrition is a prevalent issue among individuals with SCA, affecting patients of different age groups and disease severities. Nutritional deficiencies, including vitamins, minerals, and essential nutrients, are common in this population. The impact of malnutrition on disease outcomes is significant, with associations between nutrient status and complications such as pain crises, infections, and impaired quality of life. This paper also reviews nutritional interventions aimed at addressing malnutrition in SCA patients. While dietary counseling, supplementation, and personalized nutrition plans have shown promise in improving nutritional status, challenges such as patient adherence and access to healthcare must be addressed to optimize their effectiveness.

## 1. Introduction

Sickle cell anemia (SCA), a genetic hemoglobinopathy characterized by the production of abnormal hemoglobin, has been the subject of extensive research and clinical investigation. Individuals living with SCA face a myriad of challenges, from chronic pain and vaso-occlusive events to a higher risk of infections. Yet, one facet of the disease that has garnered relatively less attention but is of paramount importance is malnutrition. Malnutrition in SCA is a multifaceted concern with significant implications for both the physical and overall well-being of patients.^[1–5]^ SCA is prevalent in regions with a high prevalence of malaria, and in many of these areas, malnutrition is a concomitant issue. The disease itself, through its complex pathophysiology, introduces unique nutritional requirements and challenges, affecting the dietary patterns and nutrient status of patients. In addition, socioeconomic disparities, limited access to healthcare, and dietary restrictions compound the issue. The prevalence of malnutrition in individuals with SCA is an emerging concern that deserves a more comprehensive exploration.^[6–9]^

This paper seeks to address the critical aspects of malnutrition in SCA, including its prevalence, impact on disease outcomes, and potential interventions. The underlying pathophysiological processes of SCA, which involve chronic inflammation, oxidative stress, and hypermetabolism, elevate the nutritional needs of patients. The altered dietary habits and nutrient deficiencies, common among SCA patients, can exacerbate their vulnerability to a range of clinical complications.^[10]^ The impact of malnutrition on the SCA population is far-reaching. It is associated with an increased frequency of pain crises, more extended hospitalizations, delayed growth and development, and compromised quality of life. Despite its evident significance, malnutrition often remains under-addressed in the clinical management of SCA.^[11]^

This paper aims to fill this critical knowledge gap by presenting a comprehensive review of the prevalence and impact of malnutrition in individuals with SCA. Moreover, it delves into the existing nutritional interventions and strategies for alleviating malnutrition, thereby enhancing the holistic care and well-being of SCA patients. By shedding light on this often-overlooked aspect of the disease, we hope to advocate for greater recognition and improved management of malnutrition in SCA, ultimately contributing to better health outcomes and improved quality of life for those living with this challenging condition.

## 2. Pathophysiology of SCA and its implications for nutrition

SCA is a hereditary hemoglobinopathy characterized by the production of abnormal hemoglobin, known as hemoglobin S (HbS), instead of normal hemoglobin A. In SCA, the abnormal HbS causes red blood cells to become rigid and assume a sickle shape, leading to hemolysis (destruction of red blood cells). This results in chronic anemia. Anemia increases the body demand for nutrients, particularly iron, as iron is a key component of hemoglobin. Individuals with SCA may require higher dietary iron intake to support increased red blood cell production.^[12–16]^ Inflammation is a common feature of SCA due to ongoing hemolysis, endothelial activation, and ischemia-reperfusion injury. Chronic inflammation can lead to increased energy expenditure and protein turnover. It may be necessary to adjust dietary intake to meet these increased demands, including higher calorie and protein consumption. SCA is associated with higher levels of oxidative stress due to the release of free heme and iron from damaged red blood cells. Antioxidant nutrients such as vitamins C and E may be important to help combat oxidative stress. These nutrients can be obtained from a diet rich in fruits, vegetables, and nuts. Vaso-occlusive crises occur when the abnormally shaped red blood cells obstruct blood vessels, leading to pain, organ damage, and impaired blood flow.^[17–21]^

Proper hydration and maintenance of a balanced diet are essential to support overall health and minimize the risk of dehydration, which can exacerbate vaso-occlusive events. SCA can lead to delayed growth and development in children due to chronic anemia and nutritional deficits. Adequate nutrient intake, especially protein, is crucial for growth and development in children with SCA. Nutritional support and monitoring are essential to ensure normal growth. The increased energy expenditure and nutritional requirements in individuals with SCA result from the combined effects of anemia, inflammation, and hypermetabolism. SCA patients may require a diet that provides additional calories, protein, vitamins, and minerals to meet their heightened metabolic needs. Some SCA patients may have dietary restrictions or preferences based on cultural or medical considerations. It is essential to work with patients to create dietary plans that accommodate their specific needs, taking into account both their nutritional requirements and dietary restrictions.^[22–26]^

## 3. Malnutrition in individuals with SCA

Malnutrition in individuals with SCA is a multifaceted concern with significant implications for health and overall well-being.^[27]^ The chronic anemia associated with SCA leads to an increased demand for nutrients, particularly iron, folate, and vitamin B12. Hemolysis and the turnover of red blood cells necessitate higher dietary intake of these essential nutrients.^[28]^ Individuals with SCA often experience chronic inflammation due to the ongoing hemolysis, endothelial activation, and ischemia-reperfusion injury. Inflammatory processes can lead to increased energy expenditure and protein turnover, further elevating nutritional requirements.^[29]^ Pain crises are common in SCA and can result in loss of appetite. During these episodes, individuals may have reduced food intake, which can lead to inadequate nutrition, particularly during acute episodes of vaso-occlusive pain.^[30]^

Chronic inflammation and gastrointestinal complications can affect nutrient absorption, particularly in the intestines. Malabsorption can lead to deficiencies in essential vitamins and minerals, such as vitamin D, calcium, and magnesium.^[29]^ Proper hydration is essential for individuals with SCA to maintain blood flow and prevent vaso-occlusive events. Dehydration can exacerbate the risk of vaso-occlusive crises and can lead to further complications. Ensuring adequate fluid intake is a crucial aspect of nutritional management.^[31]^ Some individuals with SCA may adhere to dietary restrictions based on cultural practices or personal preferences. These restrictions can limit dietary choices and may contribute to nutritional deficits.^[32]^

Socioeconomic disparities can impact access to nutritious food and healthcare. Lower-income individuals may face barriers to obtaining a balanced and nutrient-rich diet. Children with SCA are at risk of growth and development delays due to chronic anemia and nutritional deficits. Nutritional support and monitoring are essential to ensure normal growth and development.^[33]^ Nutrient deficiencies can weaken the immune system, increasing the susceptibility to infections. Malnutrition can exacerbate the severity of SCA complications, such as pain crises, acute chest syndrome, and stroke.^[34]^ Nutritional deficits can impair wound healing and recovery from vaso-occlusive events or surgery. Malnutrition can contribute to fatigue, weakness, and reduced overall quality of life. Nutrient deficiencies can have cognitive and neurological effects, particularly in children, potentially impacting learning and development.^[35]^ Managing malnutrition in individuals with SCA involves a multidisciplinary approach, including healthcare providers, nutritionists, and the individuals themselves. Nutritional interventions should address specific deficiencies and adapt to the unique needs of each patient, taking into account factors such as age, disease severity, and the presence of specific complications. Regular nutritional assessments, dietary counseling, and supplementation when necessary are important components of managing malnutrition in SCA.^[36]^

## 4. Malnutrition on SCA outcomes and complications

Malnutrition in individuals with SCA can have significant consequences for health outcomes and the incidence of complications. Malnutrition, which may result from nutrient deficiencies or inadequate dietary intake, can exacerbate the severity of the disease and its associated complications.^[37]^ Malnutrition can lead to a weakened immune system and reduced pain threshold, increasing the likelihood of vaso-occlusive pain crises in SCA patients. These crises are one of the hallmark features of the disease and are often associated with severe pain, hospitalizations, and complications.^[38]^ Malnourished individuals with SCA may experience longer hospital stays during vaso-occlusive crises due to delayed recovery. Nutrient deficiencies can hinder the body ability to heal and repair damaged tissues, leading to extended hospitalizations and more extensive healthcare costs.^[28]^

Malnutrition can impede the growth and development of children with SCA. Stunted growth and delayed sexual maturation are common consequences. Delayed development can have long-term effects on the individual overall health and quality of life.^[39]^ Nutrient deficiencies, especially in essential vitamins and minerals, can weaken the immune system. This makes individuals with SCA more susceptible to infections, including those caused by bacteria and viruses, which can further complicate their health and increase the frequency of hospitalizations.^[40]^ Nutrient deficiencies, particularly vitamin B12 and folic acid, can lead to neurological complications, such as cognitive impairment and peripheral neuropathy. These complications can affect cognitive function and quality of life.^[41]^

Malnutrition can contribute to the risk of developing ACS, a potentially life-threatening complication of SCA. ACS is characterized by lung inflammation and is often triggered by infections. Weakened immune function and nutritional deficits can increase the susceptibility to infections that may lead to ACS.^[42]^ Malnutrition can exacerbate chronic anemia in SCA, leading to fatigue, weakness, and reduced exercise tolerance. This can further limit the patient overall quality of life and may lead to complications such as organ damage.^[43]^ Malnourished individuals may have a reduced response to treatment interventions, including blood transfusions or medications. Nutritional deficits can hinder the effectiveness of these therapies, making disease management more challenging.

## 5. Factors contributing to malnutrition in SCA

Several factors contribute to malnutrition in individuals with SCA, a complex genetic disorder. Understanding these factors is essential for effectively addressing malnutrition in this population. Here are some key contributors to malnutrition in individuals with SCA:

### 5.1. Increased nutritional requirements

Individuals with SCA often have higher nutritional requirements due to chronic anemia and increased turnover of red blood cells. The body needs more nutrients, particularly iron, to support red blood cell production.^[44]^

### 5.2. Chronic inflammation

Chronic inflammation is a common feature of SCA. Ongoing hemolysis (destruction of red blood cells), endothelial activation, and ischemia-reperfusion injury contribute to inflammation. This chronic inflammation increases energy expenditure and protein turnover, which can lead to a higher demand for nutrients.^[45]^

### 5.3. Pain crises and anorexia

Pain crises are a frequent complication of SCA and can result in a loss of appetite. During these painful episodes, individuals may have reduced food intake, which can lead to inadequate nutrition, particularly during acute episodes of vaso-occlusive pain.^[46]^

### 5.4. Nutrient malabsorption

Chronic inflammation and gastrointestinal complications can affect the absorption of nutrients, particularly in the intestines. Malabsorption can lead to deficiencies in essential vitamins and minerals, such as vitamin D, calcium, and magnesium.

### 5.5. Hydration and dehydration

Proper hydration is essential for individuals with SCA to maintain blood flow and prevent vaso-occlusive events. Dehydration can exacerbate the risk of vaso-occlusive crises and can lead to further complications. Ensuring adequate fluid intake is crucial for nutritional management.^[47]^

### 5.6. Cultural and dietary restrictions

Some individuals with SCA may adhere to dietary restrictions based on cultural practices or personal preferences. These restrictions can limit dietary choices and may contribute to nutritional deficits.^[32]^

### 5.7. Socioeconomic factors

Socioeconomic disparities can impact access to nutritious food and healthcare. Lower-income individuals may face barriers to obtaining a balanced diet and may lack access to healthcare resources that can help address malnutrition.

### 5.8. Growth and development delays

Children with SCA are at risk of growth and development delays due to chronic anemia and nutritional deficits. Nutritional support and monitoring are essential to ensure normal growth and development.^[48]^

### 5.9. Dietary preferences and aversions

Individuals with SCA may have dietary preferences or aversions that impact their food choices. Some may avoid specific foods due to a perceived association with pain crises or other complications.^[49]^

### 5.10. Medication side effects

Medications commonly used to manage SCA, such as hydroxyurea, may have side effects that affect appetite and nutrient absorption, potentially contributing to malnutrition.^[11]^

## 6. Prevalence of malnutrition in SCA

The prevalence of malnutrition in individuals with SCA can vary depending on factors such as geographical location, access to healthcare, socioeconomic status, and the specific nutritional status of the affected individuals. While there is no universal prevalence rate for malnutrition in SCA, research and clinical observations have highlighted its significance. The prevalence of malnutrition in SCA can vary widely among different populations and regions. In some areas with a high prevalence of both SCA and malnutrition, the risk may be greater. However, there is no fixed prevalence rate applicable to all SCA patients.^[50]^ Malnutrition can be particularly common in children and adolescents with SCA. Chronic anemia, growth and developmental delays, and increased nutritional demands during periods of growth make this population more vulnerable to malnutrition.^[51]^ Malnutrition in SCA may go underdiagnosed because it can be masked by the chronic anemia that is characteristic of the disease. The focus on managing anemia can sometimes overshadow the importance of assessing and addressing malnutrition. While not all individuals with SCA are malnourished, certain nutrient deficiencies, such as low levels of vitamin D, folate, or iron, are frequently observed in this population.

The presence of chronic health challenges, such as vaso-occlusive pain crises, frequent infections, and hospitalizations, can contribute to malnutrition. These factors can disrupt normal eating patterns and reduce nutrient intake. The prevalence of malnutrition in SCA can be better understood through screening and monitoring. Regular nutritional assessments and evaluations can help identify individuals at risk and provide opportunities for early intervention.^[52]^ Nutritional interventions, including dietary counseling, supplementation, and personalized nutrition plans, can help mitigate the effects of malnutrition. However, the success of these interventions may vary among individuals. Cultural dietary practices and preferences can influence nutritional status. Some individuals with SCA may adhere to dietary restrictions or have aversions to specific foods, which can impact their nutritional intake.

## 7. Impact of malnutrition on SCA

Malnutrition in individuals with SCA can have a significant impact on their health and well-being. SCA is a complex genetic disorder characterized by chronic anemia and a range of complications, and malnutrition can exacerbate the severity of the disease and its associated challenges.^[53]^ Malnutrition can weaken the immune system and reduce the pain threshold, leading to an increased frequency of vaso-occlusive pain crises. These crises are characterized by severe pain and can result in frequent hospitalizations and complications.^[28]^ Malnourished individuals with SCA may experience extended hospital stays during vaso-occlusive crises due to delayed recovery. Nutrient deficiencies can hinder the body ability to heal and repair damaged tissues, leading to prolonged hospitalizations and increased healthcare costs. Malnutrition can impede the growth and development of children with SCA. Stunted growth and delayed sexual maturation are common consequences, affecting the long-term health and quality of life of these individuals.^[54]^

Nutrient deficiencies, especially in essential vitamins and minerals, can weaken the immune system. Weakened immunity makes individuals with SCA more susceptible to infections, including bacterial and viral infections, which can further complicate their health and lead to frequent hospitalizations.^[54]^ Malnutrition can lead to neurological complications, particularly in the case of vitamin B12 and folic acid deficiencies. Cognitive impairment and peripheral neuropathy are potential consequences that can impact cognitive function and overall quality of life. Malnutrition can contribute to the risk of developing ACS, a potentially life-threatening complication of SCA. ACS is characterized by lung inflammation and is often triggered by infections. Malnutrition can increase susceptibility to infections that may lead to ACS.^[52]^ Malnutrition can exacerbate the chronic anemia in SCA, resulting in fatigue, weakness, and reduced exercise tolerance. These effects can limit an individual overall quality of life and may contribute to complications such as organ damage. Malnourished individuals may experience a reduced response to treatment interventions, including blood transfusions or medications. Nutritional deficits can hinder the effectiveness of these therapies, making disease management more challenging.^[28]^ The impact of malnutrition on individuals with SCA is significant and far-reaching. Addressing malnutrition through proper nutrition, dietary counseling, and nutritional interventions is essential for improving health outcomes and reducing the risk of complications. A multidisciplinary approach involving healthcare providers, nutritionists, and individuals themselves is crucial to managing malnutrition effectively and enhancing overall health and quality of life in the context of SCA.

## 8. Nutritional interventions in SCA

Nutritional interventions play a crucial role in the management of SCA. Individuals with SCA have unique nutritional needs due to chronic anemia, increased energy expenditure, and potential nutrient deficiencies. Proper nutrition can help mitigate the impact of the disease and improve overall health.^[55]^ Individuals with SCA should receive dietary counseling from registered dietitians or nutritionists who are familiar with the specific nutritional needs of SCA patients. Counseling should include information on maintaining a balanced diet, hydration, and nutrient-rich food choices.^[52]^ Folate supplementation can help support red blood cell production. SCA patients are often deficient in vitamin D, so supplementation may be recommended. In some cases, iron supplementation may be necessary, but this should be closely monitored, as excessive iron can be harmful in SCA patients.^[56]^ Adequate hydration is crucial for SCA patients to maintain blood flow and prevent vaso-occlusive events. Patients should be encouraged to drink plenty of fluids, especially during hot weather or when experiencing fever or illness.^[57]^

A well-balanced diet that includes a variety of nutrient-rich foods is essential. SCA patients should consume a diet that provides adequate calories, protein, vitamins, and minerals. Foods rich in iron, such as lean meats and dark leafy greens, can help support red blood cell production. For individuals who require iron supplementation, it is important to manage iron intake carefully. Excessive iron can lead to iron overload, which can be harmful. Monitoring iron levels through blood tests is essential. Some individuals with SCA may experience pain crises triggered by certain foods, such as those high in sodium or processed foods. Identifying and avoiding trigger foods can help manage pain and complications.

## 9. Nutritional support for growth and development

Children and adolescents with SCA may require specific nutritional support to ensure normal growth and development. Nutritionists can work with pediatric patients and their families to create age-appropriate meal plans.^[58]^ Considering cultural dietary practices and preferences is important. Nutrition interventions should be culturally sensitive and respect individual choices and traditions. Regular nutritional assessments and monitoring of nutrient levels are essential to track nutritional status and make necessary adjustments to the dietary plan. Healthcare providers, nutritionists, and patients should work together to develop and implement a personalized nutrition plan that considers the individual age, disease severity, and specific nutritional needs.^[59]^ Nutritional interventions for SCA should be tailored to the individual needs and may evolve over time. Regular communication with healthcare providers and nutrition experts is essential to ensure that the nutritional plan is effective and well-suited to the changing needs of individuals with SCA. Proper nutrition is an integral part of managing SCA and can significantly impact the health and well-being of those living with the condition.^[28]^

## 10. Challenges and barriers of malnutrition in SCA

Addressing malnutrition in individuals with SCA is essential for improving health outcomes, but several challenges and barriers can complicate this process. These challenges may be influenced by factors such as healthcare access, socioeconomic status, cultural considerations, and the complex nature of the disease itself. Limited awareness and understanding of the nutritional needs of individuals with SCA can lead to delayed or inadequate interventions. Healthcare providers, patients, and caregivers may not be fully informed about the importance of nutrition in managing SCA.^[60]^

Socioeconomic disparities and lack of access to healthcare can impede the ability of individuals with SCA to receive proper nutritional assessments and interventions. Some individuals may face barriers to accessing healthcare resources and expertise. Cultural dietary practices and preferences can influence nutritional choices. Dietary restrictions or aversions based on cultural or personal beliefs can limit the variety of foods available to individuals with SCA, potentially leading to nutritional deficits. Meeting the complex nutritional requirements of individuals with SCA, including addressing chronic anemia and inflammation, can be challenging. Nutritional management may require personalized plans and careful consideration of specific needs.^[61]^ SCA is associated with chronic pain crises, frequent infections, and hospitalizations. These health challenges can disrupt normal eating patterns, leading to reduced nutrient intake and making it difficult to manage malnutrition.^[20]^

Some individuals with SCA may experience pain crises triggered by specific foods. Avoiding these foods can lead to dietary restrictions and challenges in maintaining a balanced diet.^[62]^ Living with SCA can be emotionally and psychologically challenging. Mental health factors, such as depression or anxiety, can affect eating habits and overall well-being, potentially contributing to malnutrition.^[63]^ In regions with limited resources, individuals with SCA may not have access to nutritional support, such as dietary counseling or nutritional supplements. This lack of resources can hinder effective malnutrition management.^[64]^ Patient adherence to nutritional recommendations can be a barrier. Individuals may struggle to follow dietary plans or take prescribed supplements consistently, impacting the effectiveness of nutritional interventions. The severity of SCA can vary among individuals. Managing malnutrition in severe cases may be more challenging and require more intensive interventions.^[59]^

## 11. Nutrition screening tool in sickle disease in children and adults

Individuals with sickle cell disease (SCD) often face unique challenges related to their nutritional status, which necessitates the development and utilization of effective nutrition screening tools.^[65]^ These tools are designed to identify individuals at risk of malnutrition, assess their dietary habits, and guide healthcare professionals in providing targeted interventions. Implementing a nutrition screening tool for both children and adults with SCD is crucial to ensure that their nutritional needs are addressed comprehensively. A robust nutrition screening tool for SCD should encompass various components. It should consider the disease-specific factors such as the frequency and severity of pain crises, medication impact on appetite, and any dietary restrictions associated with the condition. Additionally, assessing general health status, energy levels, and appetite provides insights into overall well-being.^[66]^ Dietary habits, including hydration, regularity of meals, and intake of essential nutrients, should be thoroughly examined. A comprehensive screening tool must also account for the potential impact of coexisting conditions on nutritional status, acknowledging the complexity of healthcare needs in individuals with SCD.^[67]^

Given that SCD affects both children and adults, an effective nutrition screening tool should be adaptable across different age groups.^[67]^ Children may have distinct nutritional requirements due to growth and development, while adults may face challenges related to chronic pain and increased nutrient demands during crises. A tool that considers these age-specific factors ensures a more accurate assessment of nutritional status and facilitates targeted interventions tailored to the unique needs of each population. In children with SCA, growth and development are particularly critical considerations. Nutrition screening tools tailored for pediatric populations should address age-appropriate nutritional requirements, growth patterns, and the potential impact of SCD on developmental milestones. For adults, the emphasis may shift toward managing chronic complications, maintaining a healthy weight, and addressing nutrient deficiencies associated with the disease.

The implementation of a nutrition screening tool in the clinical care of individuals with SCD holds promise for improving their overall health outcomes.^[68]^ Regular screening can lead to early identification of nutritional issues, allowing for timely interventions such as dietary counseling, supplementation, or collaboration with registered dietitians. As research in the field of SCD continues to evolve, future iterations of nutrition screening tools should incorporate advancements in our understanding of the disease and its impact on nutritional status, ensuring that they remain effective in guiding patient care. The nutritional status of individuals with SCD is crucial for managing the overall health and well-being of both children and adults. Various nutrition screening tools have been developed to assess the specific needs and challenges associated with SCD.^[69]^ One such tool is the Malnutrition Universal Screening Tool (MUST), which is commonly used in diverse populations but may need adaptation for the unique considerations of SCA.^[70]^ Malnutrition Universal Screening Tool (MUST), or any similar tool, can serve as a valuable asset in identifying individuals at risk of malnutrition in the context of SCD. For children and adults alike, these screening tools typically encompass a range of parameters, including overall health, energy levels, appetite, sickle cell crisis frequency, pain levels, and dietary habits. These tools provide a comprehensive evaluation, considering disease-specific factors such as medication usage, dietary restrictions, and the impact of SCD on nutritional intake.^[69]^

## 12. Future directions of malnutrition in SCA

The management of malnutrition in individuals with SCA is an evolving field with ongoing research and potential future directions. Tailoring nutrition plans to individual SCA patients based on their age, disease severity, and specific nutritional needs is an area that warrants further research. Personalized plans can optimize the management of malnutrition and improve health outcomes.^[71]^ Research into precision medicine, including genetic and molecular profiling, may provide insights into the specific nutritional requirements of SCA patients. Precision approaches can help identify genetic factors that influence nutrient metabolism and guide personalized nutritional interventions. Studying the interaction between an individual genetic makeup and their response to specific nutrients is an emerging field. Understanding how genetic variations affect nutrient utilization in SCA can lead to more targeted nutritional interventions. The development of innovative dietary supplements, such as fortified foods or micronutrient-rich products, designed specifically for individuals with SCA, could address common nutrient deficiencies and enhance overall nutrition. Leveraging telehealth and telemedicine platforms to provide nutritional counseling and support for individuals with SCA, especially in regions with limited access to healthcare, can be a promising direction. These technologies can improve access to nutritional expertise and ongoing monitoring. Conducting clinical trials and intervention studies to assess the effectiveness of different nutritional approaches in managing malnutrition in SCA is essential. These studies can provide evidence-based guidelines for nutrition interventions.

Exploring the impact of behavioral interventions, such as motivational counseling, on dietary adherence and nutritional status can help individuals with SCA develop better eating habits and improve their overall health. The use of telehealth and telemedicine can improve access to nutritional counseling and support for individuals with SCA, especially in regions with limited access to healthcare resources. Developing collaborative care models that involve a multidisciplinary team of healthcare providers, nutritionists, and psychologists can address the complex nutritional and psychosocial needs of individuals with SCA. Implementing global health initiatives to raise awareness and improve the nutritional management of SCA, especially in regions with a high prevalence of the disease, can have a substantial impact on reducing malnutrition. Empowering individuals with SCA and their caregivers through education about the importance of nutrition in disease management and providing them with practical tools to make informed dietary choices. Conducting longitudinal studies to assess the long-term effects of nutritional interventions on health outcomes and complications in individuals with SCA.

These future directions represent opportunities to enhance the nutritional management of SCA and improve the overall well-being and quality of life for individuals living with this complex condition. Research, innovation, and collaboration among healthcare professionals, researchers, and patients will continue to shape the field of nutrition in SCA.

## 13. Conclusion

The management of malnutrition in individuals with SCA is a critical aspect of comprehensive care that cannot be overlooked. Malnutrition, while often underrecognized, exerts a profound impact on the health and well-being of those living with SCA. This publication has shed light on the prevalence of malnutrition, its impact on disease outcomes, and potential interventions. The prevalence of malnutrition in SCA varies, influenced by factors such as geography, healthcare access, and socioeconomic status. However, it is clear that malnutrition affects a substantial portion of the SCA population, especially children and adolescents, and it is associated with a host of complications and challenges. Malnutrition in SCA is not a standalone issue but an interconnected aspect of the complex pathophysiology of the disease. Chronic anemia, inflammation, and hypermetabolism necessitate increased nutritional requirements, while factors such as pain crises, nutrient malabsorption, and dietary restrictions hinder adequate nutrition. The consequences of malnutrition are far-reaching, impacting pain crises, hospitalizations, growth and development, immune function, and overall quality of life.

To address malnutrition in SCA, a multifaceted approach is necessary. Nutritional interventions, including dietary counseling, supplementation, and personalized nutrition plans, should be tailored to the specific needs of each individual. Collaborative care involving healthcare providers, nutritionists, and patients is crucial for effective management. Looking ahead, the future of malnutrition management in SCA holds promise. Personalized nutrition plans, precision medicine, innovative dietary supplements, and the integration of telehealth and telemedicine can transform the way we address malnutrition. Behavioral interventions and global health initiatives can further empower individuals with SCA to take charge of their nutritional well-being.

## Author contributions

**Conceptualization:** Emmanuel Ifeanyi Obeagu.

**Methodology:** Emmanuel Ifeanyi Obeagu, Getrude Uzoma Obeagu.

**Supervision:** Emmanuel Ifeanyi Obeagu.

**Visualization:** Emmanuel Ifeanyi Obeagu.

**Writing—original draft:** Emmanuel Ifeanyi Obeagu, Getrude Uzoma Obeagu.

**Writing—review & editing:** Emmanuel Ifeanyi Obeagu, Getrude Uzoma Obeagu.

## References

[1] Obeagu EI Ochei KC Nwachukwu BN. Sickle cell anaemia: a review. Sch J Appl Med Sci. 2015;3:2244–52.

[2] Obeagu EI. Erythropoeitin in sickle cell anaemia: a review. Int J Res Stud Med Health Sci. 2020;5:22–8.

[3] Obeagu EI. Sickle cell anaemia: haemolysis and anemia. Int J Curr Res Chem Pharm Sci. 2018;5:20–1.

[4] Obeagu EI Muhimbura E Kagenderezo BP. An update on interferon gamma and C reactive proteins in sickle cell anaemia crisis. J Biomed Sci. 2022;11:84.

[5] Obeagu EI Bunu UO Obeagu GU. Antioxidants in the management of sickle cell anaemia: an area to be exploited for the wellbeing of the patients. Int Res Med Health Sci. 2023;6:12–7.

[6] Wegmüller R Bentil H Wirth JP. Anemia, micronutrient deficiencies, malaria, hemoglobinopathies and malnutrition in young children and non-pregnant women in Ghana: findings from a national survey. PLoS One. 2020;15:e0228258. 31999737 10.1371/journal.pone.0228258 PMC6991996

[7] Obeagu EI Ogunnaya FU Obeagu GU. Sickle cell anaemia: a gestational enigma. Eur J Biomed Pharm Sci. 2023;10:72–5.

[8] Obeagu EI. An update on micro RNA in sickle cell disease. Int J Adv Res Biol Sci. 2018;5:157–8.

[9] Obeagu EI Babar Q. Covid-19 and sickle cell anemia: susceptibility and severity. J Clin Lab Res. 2021;3:2768–0487.

[10] Kamal S Naghib MM Al Zahrani J. Influence of nutrition on disease severity and health-related quality of life in adults with sickle cell disease: a prospective study. Mediterr J Hematol Infect Dis. 2021;13. 10.4084/MJHID.2021.007 PMC7813275 33489046

[11] Abdullahi SU Gambo S Murtala HA. Feasibility trial for the management of severe acute malnutrition in older children with sickle cell anemia in Nigeria. Blood Adv. 2023;7:6024–34. 37428866 10.1182/bloodadvances.2023010789 PMC10582275

[12] Obeagu EI. Depression in sickle cell anemia: an overlooked battle. Int J Curr Res Chem Pharm Sci. 2023;10:41.

[13] Obeagu EI Obeagu GU. Evaluation of hematological parameters of sickle cell anemia patients with osteomyelitis in a tertiary hospital in Enugu, Nigeria. J Clin Lab Res. 2023;6:2768–0487.

[14] Obeagu EI Dahir FS Francisca U. Hyperthyroidism in sickle cell anaemia. Int J Adv Res Biol Sci. 2023;10:81–9.

[15] Obeagu EI Obeagu GU Akinleye CA. Nosocomial infections in sickle cell anemia patients: prevention through multi-disciplinary approach: a review. Medicine (Baltimore). 2023;102:e36462. 38050205 10.1097/MD.0000000000036462 PMC10695528

[16] Njar VE Ogunnaya FU Obeagu EI. Knowledge and prevalence of the sickle cell trait among undergraduate students of the University of Calabar. Prevalence. 2023;5:0–5.

[17] Swem CA Ukaejiofo EO Obeagu EI. Expression of micro RNA 144 in sickle cell disease. Int J Curr Res Med Sci. 2018;4:26–32.

[18] Obeagu EI Nimo OM Bunu UO. Anaemia in children under five years: African perspectives. Int J Curr Res Biol Med. 2023;1:1–7.

[19] Obeagu EI. Sickle cell anaemia: historical perspective, pathophysiology and clinical manifestations. Int J Curr Res Chem Pharm Sci. 2018;5:13–5.

[20] Obeagu EI Obeagu GU. Sickle cell anaemia in pregnancy: a review. Int Res Med Health Sci. 2023;6:10–3.

[21] Obeagu EI Mohamod AH. An update on Iron deficiency anaemia among children with congenital heart disease. Int J Curr Res Chem Pharm Sci. 2023;10:45–8.

[22] Edward U Osuorji VC Nnodim J. Evaluation of trace elements in sickle cell anaemia patients attending imo state specialist hospital, Owerri. Madonna Univ J Med Health Sci. 2022;2:218–34.

[23] Buhari HA Ahmad AS Obeagu EI. Current advances in the diagnosis and treatment of sickle cell anaemia. Appl Sci (Nijbas). 2023;4.

[24] Nnodim J Uche U Ifeoma U. Hepcidin and erythropoietin level in sickle cell disease. Br J Med Med Res. 2015;8:261–5.

[25] Aloh GS Obeagu EI Okoroiwu IL. Antioxidant-mediated heinz bodies levels of sickle erythrocytes under drug-induced oxidative stress. Eur J Biomed Pharm Sci. 2015;2:502–7.

[26] Obeagu EI Malot S Obeagu GU. HIV resistance in patients with sickle cell anaemia. Newport Int J Sci Exp Sci (NIJSES). 2023;3:56–9.

[27] Mawson AR. Health Disparities and the Ancestral Environment: An Evolutionary Perspective. Cambridge Scholars Publishing; 2023.

[28] Khan SA Damanhouri G Ali A. Precipitating factors and targeted therapies in combating the perils of sickle cell disease---a special nutritional consideration. Nutr Metab. 2016;13:1–2. 10.1186/s12986-016-0109-7 PMC4977632 27508000

[29] Guarda CC Santiago RP Fiuza LM. Heme-mediated cell activation: the inflammatory puzzle of sickle cell anemia. Expert Rev Hematol. 2017;10:533–41. 28482712 10.1080/17474086.2017.1327809

[30] Yousif OO Hassan MA Al-Naama LM. Red blood cell and serum magnesium levels among children and adolescents with sickle cell anemia. Biol Trace Elem Res. 2018;186:295–304. 29637408 10.1007/s12011-018-1307-0

[31] Piccin A Murphy C Eakins E. Insight into the complex pathophysiology of sickle cell anaemia and possible treatment. Eur J Haematol. 2019;102:319–30. 30664257 10.1111/ejh.13212

[32] Roberts KJ Binns HJ Vincent C. A scoping review: family and child perspectives of clinic-based obesity treatment. J Pediatr Nurs. 2021;57:56–72. 33271477 10.1016/j.pedn.2020.10.025 PMC7946710

[33] Chikani UN Bisi-Onyemaechi A Ohuche I. The effect of sickle cell anemia on the linear growth of Nigerian children. J Pediatr Endocrinol Metab. 2021;34:1283–90. 34271599 10.1515/jpem-2021-0232

[34] Gombart AF Pierre A Maggini S. A review of micronutrients and the immune system–working in harmony to reduce the risk of infection. Nutrients. 2020;12:236. 31963293 10.3390/nu12010236 PMC7019735

[35] Prevost R Feugueur G Moizan H. Management of patients with sickle cell disease in oral surgery. Literature review and update. J Stomatol Oral Maxillofac Surg. 2018;119:493–7. 29960012 10.1016/j.jormas.2018.06.010

[36] Gabrovec B Antoniadou E Soleymani D. Need for comprehensive management of frailty at an individual level: European perspective from the advantage joint action on frailty. J Rehabil Med. 2020;52:1–6. 10.2340/16501977-2687 32399576

[37] Jesus AC Konstantyner T Lôbo IK. Socioeconomic and nutritional characteristics of children and adolescents with sickle cell anemia: a systematic review. Rev Paul Pediatr. 2018;36:491–9. 30540112 10.1590/1984-0462/;2018;36;4;00010 PMC6322809

[38] Ballas SK Darbari DS. Review/overview of pain in sickle cell disease. Complement Ther Med. 2020;49:102327. 32147066 10.1016/j.ctim.2020.102327

[39] Hyacinth HI Adekeye OA Yilgwan CS. Malnutrition in sickle cell anemia: implications for infection, growth, and maturation. J Soc Behav Health Sci. 2013;7. 10.5590/JSBHS.2013.07.1.02 PMC3848498 24312698

[40] Calder PC Carr AC Gombart AF. Optimal nutritional status for a well-functioning immune system is an important factor to protect against viral infections. Nutrients. 2020;12:1181. 32756516 10.3390/nu12082326 PMC7469053

[41] Kumar N. Nutrients and neurology. Continuum (Minneap Minn). 2017;23:822–61. 28570331 10.1212/01.CON.0000520630.69195.90

[42] Williams TN Thein SL. Sickle cell anemia and its phenotypes. Annu Rev Genomics Hum Genet. 2018;19:113–47. 29641911 10.1146/annurev-genom-083117-021320 PMC7613509

[43] Broadway-Duren JB Klaassen H. Anemias. Crit Care Nurs Clin North Am. 2013;25:411–26, v. 24267278 10.1016/j.ccell.2013.09.004

[44] Nnachi OC Orih MC Edenya OO. Micronutrient levels and haemato-biochemical status of patients with sickle cell anaemia at a tertiary hospital in Abakaliki, south-eastern Nigeria: a cross-sectional study. Pan Afr Med J. 2022;41:303. 35855023 10.11604/pamj.2022.41.303.31728 PMC9250682

[45] Bandeira IC Rocha LB Barbosa MC. Chronic inflammatory state in sickle cell anemia patients is associated with HBB* S haplotype. Cytokine. 2014;65:217–21. 24290434 10.1016/j.cyto.2013.10.009

[46] Cieri-Hutcherson NE Hutcherson TC Conway-Habes EE. Systematic review of L-glutamine for prevention of vaso-occlusive pain crisis in patients with sickle cell disease. Pharmacotherapy. 2019;39:1095–104. 31505045 10.1002/phar.2329

[47] Nader E Skinner S Romana M. Blood rheology: key parameters, impact on blood flow, role in sickle cell disease and effects of exercise. Front Physiol. 2019;10:1329. 31749708 10.3389/fphys.2019.01329 PMC6842957

[48] Ukoha OM Emodi IJ Ikefuna AN. Comparative study of nutritional status of children and adolescents with sickle cell anemia in Enugu, Southeast Nigeria. Niger J Clin Pract. 2020;23:1079–86. 32788485 10.4103/njcp.njcp_476_19

[49] Acquisti A Adjerid I Balebako R. Nudges for privacy and security: Understanding and assisting users’ choices online. ACM Comput Surv. 2017;50:1–41.

[50] Ghafuri DL Abdullahi SU Galadanci NA. High prevalence of severe acute malnutrition in children with sickle cell anemia over 5 years of age in Northern Nigeria. Blood. 2017;130:127.

[51] Esezobor CI Akintan P Akinsulie A. Wasting and stunting are still prevalent in children with sickle cell anaemia in Lagos, Nigeria. Ital J Pediatr. 2016;42:1–8. 27146866 10.1186/s13052-016-0257-4 PMC4857256

[52] Singh A. Childhood malnutrition in India. In: Perspective of Recent advances in acute diarrhea. London; 2020.

[53] Essien EA Winter-Eteng BF Onukogu CU. Psychosocial challenges of persons with sickle cell anemia: a narrative review. Medicine (Baltimore). 2023;102:e36147. 38013366 10.1097/MD.0000000000036147 PMC10681612

[54] dos Santos SA de Oliveira CL Cortez PI. Nutritional status of children and adolescents with sickle cell disease. J Nutr Med Diet Care. 2018;4:1–1.

[55] Umeakunne K Hibbert JM. Nutrition in sickle cell disease: recent insights. Nutr Diet Suppl. 2019;11:9–17.

[56] Dos Santos TE De Sousa GF Barbosa MC. The role of iron overload on oxidative stress in sickle cell anemia. Biomarkers Med. 2012;6:813–9. 10.2217/bmm.12.71 23227847

[57] Osunkwo I Manwani D Kanter J. Current and novel therapies for the prevention of vaso-occlusive crisis in sickle cell disease. Ther Adv Hematol. 2020;11:2040620720955000. 33062233 10.1177/2040620720955000 PMC7534097

[58] Williams R Olivi S Li CS. Oral glutamine supplementation decreases resting energy expenditure in children and adolescents with sickle cell anemia. J Pediatr Hematol Oncol. 2004;26:619–25. 10.1097/01.mph.0000140651.65591.b8 27811601

[59] Dale JC Cochran CJ Roy L. Health-related quality of life in children and adolescents with sickle cell disease. J Pediatr Health Care. 2011;25:208–15. 21700135 10.1016/j.pedhc.2009.12.006 PMC3124665

[60] Egesa WI Nakalema G Waibi WM. Sickle cell disease in children and adolescents: a review of the historical, clinical, and public health perspective of sub-saharan africa and beyond. Int J Pediatr. 2022;2022:3885979. 36254264 10.1155/2022/3885979 PMC9569228

[61] Obeagu EI Bot YS Opoku D. Sickle cell anaemia: current burden in Africa. Int J Innov Appl Res. 2023;11:12–4.

[62] Obeagu EI Ogbuabor BN Ikechukwu OA. Haematological parameters among sickle cell anemia patients’ state and haemoglobin genotype AA individuals at Michael Okpara University of Agriculture, Umudike, Abia State, Nigeria. Int J Curr Microbiol Appl Sci. 2014;3:1000–5.

[63] Obeagu EI Opoku D Obeagu GU. Burden of nutritional anaemia in Africa: a review. Int J Adv Res Biol Sci. 2023;10:160–3.

[64] Ifeanyi E. Erythropoietin (Epo) Level in Sickle Cell Anaemia (HbSS) With falciparum malaria infection in university health services, Michael Okpara University of Agriculture, Umudike, Abia State, Nigeria. Paripex Indian J Res. 2015;4:258–9.

[65] Odame I Jain D. Sickle cell disease: progress made & challenges ahead. Indian J Med Res. 2020;151:505–8. 32719221 10.4103/ijmr.IJMR_2064_20 PMC7602930

[66] Howard J Thein SL. Optimal disease management and health monitoring in adults with sickle cell disease. Hematology Am Soc Hematol Educ Program. 2019;2019:505–12. 31808832 10.1182/hematology.2019000055 PMC6913450

[67] Oyedeji CI Hall K Luciano A. The sickle cell disease functional assessment (SCD-FA) tool: a feasibility pilot study. Pilot Feasibility Stud. 2022;8:53. 35246265 10.1186/s40814-022-01005-3 PMC8895638

[68] Bell V Varzakas T Psaltopoulou T. Sickle cell disease update: new treatments and challenging nutritional interventions. Nutrients. 2024;16:258. 38257151 10.3390/nu16020258 PMC10820494

[69] Colombatti R Hegemann I Medici M. Systematic literature review shows gaps in data on global prevalence and birth prevalence of sickle cell disease and sickle cell trait: call for action to scale up and harmonize data collection. J Clin Med. 2023;12:5538. 37685604 10.3390/jcm12175538 PMC10488271

[70] Elia M. Screening for malnutrition: a multidisciplinary responsibility. development and use of the “Malnutrition Universal Screening Tool” (“MUST”) for adults. Malnutrition Advisory Group, a Standing Committee of BAPEN. Redditch: BAPEN, Available at: https://www.bapen.org.uk/pdfs/must/must-report.pdf. 2003.

[71] Carnethon MR Pu J Howard G. Cardiovascular health in African Americans: a scientific statement from the American Heart Association. Circulation. 2017;136:e393–423. 29061565 10.1161/CIR.0000000000000534

